# Improving recycling sorting behaviour with human eye nudges

**DOI:** 10.1038/s41598-023-37019-x

**Published:** 2023-06-22

**Authors:** Lorenzo Lotti, Lory Barile, Giovanni Manfredi

**Affiliations:** 1 Institute for Sustainable Resources, The Bartlett School of Environment, Energy and Resources, University College London, 14 Upper Woburn Pl, London, WC1H 0NN UK; 2 Department of Economics, University of Warwick, Coventry, CV4 7AL UK

**Keywords:** Behavioural ecology, Climate-change ecology, Environmental economics, Neuroscience, Psychology

## Abstract

This paper tests whether visual nudges help direct attention towards existing instructions designed to increase waste sorting accuracy. The study was conducted in a quasi-experimental setting over a period of 9 weeks in two buildings of a large UK university campus. Two treatments on recycling behaviour were tested against a control group: one considered the impact of visual nudges in the form of human eyes; the other one combined human eye with pre-existing sorting instructions. Results show that for mixed recycling the combination of visual and information nudges decreased sorting errors by 7 percentage points. In contrast, visual nudges alone increased sorting errors by 4.5 percentage points. These findings indicate that, when combined, information and a visual nudge are cost-effective tools to significantly improve waste sorting behaviour. This paper adds to existing experimental evidence based on neuroscientific theories.

## Introduction

The importance of recycling stems from the very well recognised and accepted policy target of promoting actions to reduce damage to the environment. In fact, recycling prolongs products’ life and saves on the emissions associated with their production process (Linder et al*.*^1^). The effects of not recycling can be very costly to economies. Considering waste bins, every item that does not get recycled properly increases the volume of garbage being re-directed to incinerators or worst still landfill.

Despite the positive impact recycling may have on our environment and existing legislation (e.g. Waste Framework Directive) to foster it, recent reports from the EU Commission show that the volume of municipal waste has increased over the last decade, with low recycling rates and lower quality of waste (accuracy/cleanliness). Although this can partially be due to inefficient waste collection systems, more needs to be done to improve individuals’ recycling sorting behaviour. Like other sectors (Recent statistics on the UK recycling rate for waste from households (Defra^2^) show a worrying decrease to 44.4% in 2020 from 46% in 2019), recycling rates in the UK higher education (HE) record a declining recycling rate from 80% in 2019/2020 to 70% in 2020/2021. This data is concerning especially considering that universities continue to produce 250,000 tonnes of waste per year^3^. Given the role that universities play to educate and change behaviour in our next generation of leaders, exploring the impact of different waste recycling interventions on a university site represents a unique opportunity to understand how HE institutions may help developing ways to live more sustainably.

Behavioural economics literature assumes that individuals exhibit ‘bounded rationality, bounded self-will and bounded self-interest’ (see Mullainathan and Thaler^4^). For this type of individuals, the evidence suggests that a nudge might be more effective than a ‘shove’ to increase individuals’ willingness to recycle the more individuals are motivated by the intrinsic value of action (see Barile et al.^5^) (A nudge is a behavioural economic tool introduced in our choice architecture able to alter individuals’ behaviour without affecting one’s freedom to choose (Sunstein^6^)). This is particularly relevant when nudges create a cognitive shortcut, reducing individuals’ required attention to complete a task. Indeed, attention is a limited resource (Tversky and Kahneman^7^). The objective of this analysis is to provide further evidence on the efficacy of nudging tools to increase recycling behaviour as well as improve the quality of sorted waste. To this end, the study utilises a visual nudge – i.e. a picture of human eyes – and actual behaviour rather than focusing on recycling beliefs, attitudes, or intentions.

Subtle cues of observability are seen as successful (and cost-effective) tools to foster compliance in many different domains (such as littering, and bicycle theft), being able to internalise implicit injunctive norms mainly by means of reputational or surveillance effects (Gangl et al.^8^). This literature complements a wide variety of studies analysing the relevance of social norms for pro-social behaviour (see e.g. Burnham and Hare^9^; and Cialdini et al*.*^10^).

Our eyes represent strong means of communication and are often used in social situations for emotional detection. Neuroscience literature suggests that eyes’ proximity to one person impacts the involuntary neuronal system in the amygdala, the prefrontal cortex, and the superior temporal sulcus (STS), activating human behaviour and economic decision-making processes (Burnham and Hare^9^; van der Wel et al.^11^). Within the STS there are specific sub-cells responsible for identifying certain features within our visual range such as eyes, nose, mouth, cheeks, and head direction: among those cells, 64% are responsible for eye recognition (de Vries and Baldauf^12^; Emery^13^). As a result, and not surprisingly, visual cues (in the form of stylized eyespots) may have a positive impact on cooperation and pro-social behaviour and tend to increase individuals’ generosity in dictator games (see e.g. Haley and Fessler^14^). Similarly, they may be a strong driver of individuals’ willingness to contribute to public goods (see Bateson et al*.*^15^).

When watched by someone, we can feel that our reputation is under scrutiny. The effect of reputation-based interventions (watching eyes) on individuals’ injunctive norms has been extensively explored in field experiments aimed at reducing littering (see e.g. Ernest-Jones et al*.*^16^; and Francey and Bergmuller^17^), which, together with fostering recycling behaviour, is seen as an important factor in helping to tackle climate change (see Wijkman and Skånberg^18^).

In general, this literature suggests a positive relationship between relatively simple visual nudges and human social behaviour. Bateson et al*.*^19^, also shows that watching eyes can trigger people’s injunctive norms, even when descriptive norms contradict the injunctive one (Injunctive norms outline what ought to be done, and the moral action that individuals should take (Nicolls et al*.*^20^). Descriptive norms describe other individuals’ behaviours, and what is considered typical or normal, through evidence of what individuals are doing, often used as a heuristic shortcut (Ayal et al*.*^21^)). More recently, Gangl et al*.*^8^ in a large, randomised control trial (RCT) in Vienna, test the effect of different interventions (e.g. monetary information, a depicted injunctive norm, watching eyes and a nature picture) and conclude that behavioural economic tools based on implicit and soft appeals to reputation and ecology are more effective in fostering clean environments than classical interventions providing explicit information on finances and norms.

Most of these studies suggest watching eyes are a powerful instrument to induce feelings of being monitored and reputational concerns, thus increasing norm-compliant behaviour (see e.g. Ernest-Jones et al.^16^; Francey and Bergmüller^17^) or, slightly different, they suggest that the presence of visual nudges may increase feelings of surveillance and therefore help reducing non-compliant behaviours (e.g. Bateson et al.^19^). However, less is known on the role of human eyes on re-directing attention to written and easy to grasp instructions, which will be the objective of this analysis. Based on the existing evidence on littering, the positive effect of eye images on human cooperative behaviour results from reputational concerns rather than their drawing attention to written/verbal instructions, when these are made available to decision makers. Written recycling instructions can themselves be an effective means of increasing compliance with cooperative norms (Burgess et al.^22^, Durdan et al*.*^23^, Thaler and Sunstein1^24^). Informational programmes have proved to be effective to foster recycling participation (see e.g. Iyer and Kashyap^25^, and Vicente and Reis^26^). However, individuals think fast, use their gut feelings to act, and are incredibly prone to mistakes.

Therefore, in this study, we wish to better understand the direct link between cues of being observed, and the activation of motivation to comply with a ‘social’ norm when eye images are paired with written instructions. In particular, can the eye-nudge improve a complex behaviour, such as sorting recycling, when combined with pre-existing sorting instructions? Further, does the eye-nudge work by attracting attention towards the instruction or by its own produced effect of the injunctive norm? If eyes only enhance recycling behaviour when they are on a poster pointing out the written instructions, then the first, attentional interpretation of results is supported. If the eyes increase recycling behaviour even when displayed on posters without written instructions, then the second interpretation of a more direct link between cues of observation and the motivation to cooperate seems plausible. Finally, is the effect of the eye-nudge short-lived? To test these hypotheses, we conduct a field experiment and analyse recycling behaviour under two different treatments, using eye images alone versus a treatment where eye images combined with pre-existing written instructions are employed to enhance recycling. Data collected in these treatments is then compared with that obtained from a control group that did not receive any treatment. Finally, to show potential patterns of how the treatment was more efficient with certain error typologies this study adopted heatmaps, with a scale of different colours to graphically describe the changes in sorting errors.

## Methodology

The aim of this paper is to examine the relative effectiveness of two interventions in promoting individuals’ recycling behaviour: watching eyes alone and watching eyes combined with (visual and easy-to-grasp written) recycling instructions. Our main goal is to explore whether these interventions are effective at all in promoting compliance to sort waste appropriately. The experiment was conducted in two buildings of a large university campus, where access was limited to students and (academic, and professional support services (PSS) members of) staff (Hereafter, we will refer more generally to PSS and academics as staff members). The research was conducted in accordance with the relevant university guidelines and regulations, and permission to access and perform the experiment was granted by Estates. Ethics approval was also granted to conduct the research.

### Settings and participants

For simplicity, we will identify the two buildings used for the experiment as “CH” and “GS”. CH represents the control group. Here data was collected from six different floors, for a total of nine receptacles. The treatments were tested at GS, where data was collected from fourteen receptacles on six different floors. Treatment 1 was tested on nine receptacles from floor one to floor four. Treatment 2 was tested on five receptacles, on floors five and six.

As a field experiment, we did not have direct influence over the allocation of participants into treatment and control groups. However, it is worth mentioning that in CH some floors were accessible only to members of staff and PhD students (both grouped as “staff members” in the analysis below). These are floors one, three and four. All other areas were opened to students and staff. Although, in GS there was no limited floor access, we recognise that the floor architecture may have an impact on participants accessibility and traffic (To give an example, in GS some floors are specifically designed for students, with facilities that may only be appealing to them (such as open study spaces)). We control for this in the empirical analysis below. We also record extra-trial factors such as proximity to other general waste bins, to check whether this influenced individuals’ recycling behaviour. Table 1 (see section “Defining sorting errors” below) depicts receptacles’ characteristics by buildings, floors, locations (All locations were in proximity to rinsing facilities except for the location “room”. However, due to the small number of bins located in the basement (two) and the fact that this is a low traffic area, we believe that lack of this does not have a major impact on our results), participants, and extra-trial factors.

**Table 1:** List of receptacles, by buildings, location, participants, and extra-trial factors.

Building		Floor	Location	Participants	Extra-trial factors
Control group	CH	− 1	Room	Students	MR and NR
		0	Room	Mixed	MR
		0	Hallway	Mixed	MR
		0	Kitchen	Students	NR
		0	Library	Students	None
		1	Kitchen	Staff	NR
		2	Kitchen	Students	None
		3	Kitchen	Staff	None
		4	Kitchen	Staff	None
		1	Hallway	Mixed	DR
		1	Kitchen/corridor	Staff	MR
		1	Kitchen/corridor	Staff	None
		2	Hallway	Mixed	None
Treatment group 1	GS	2	Kitchen/corridor	Students	MR and DR
		3	Hallway	Mixed	None
		3	Kitchen/corridor	Students	None
		4	Hallway	Mixed	None
		4	Kitchen/corridor	Students	2 MR and DR
		5	Hallway	Mixed	None
		5	Kitchen/corridor	Students	2 MR and DR
Treatment group 2	GS	6	Hallway	Mixed	None
		6	Kitchen/corridor	Students	2 MR
		6	Kitchen/corridor	Students	None

The table reports receptacles’ characteristics by buildings, location, participants, and extra-trial factors. The top panel of the table reports the characteristics of the 9 receptacles in the control group. The middle panel of the table considers the 9 receptacles in treatment 1 and the remaining 5 receptacles are reported at the bottom of the table. Floor “ − 1” refers to the basement floor, and “0” represents the ground floor. Location “Kitchen/corridor” refers to bins located in corridors in kitchen proximity, whereas “Kitchen” refers to receptacles located within kitchens. “Mixed” participants indicate areas opened to staff and students. “MR” is mixed-recycling, and “DR” and “NR” refer to dry-recycling and non-recycling, respectively.

### Sample, experimental process and manipulation of interventions

**Figure 1:**
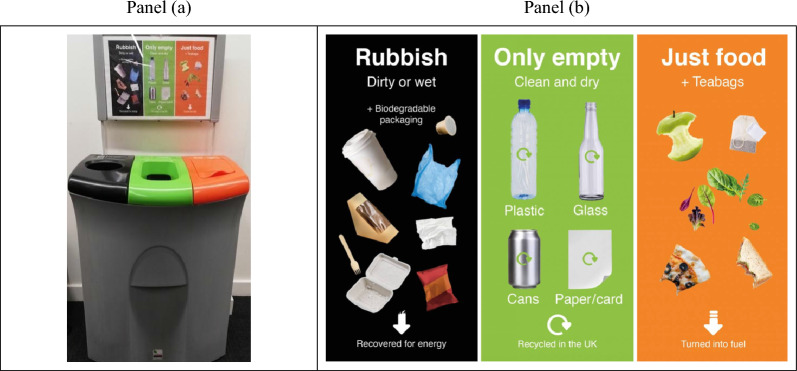
Bins receptacle and recycling instructions.

**Figure 2:**
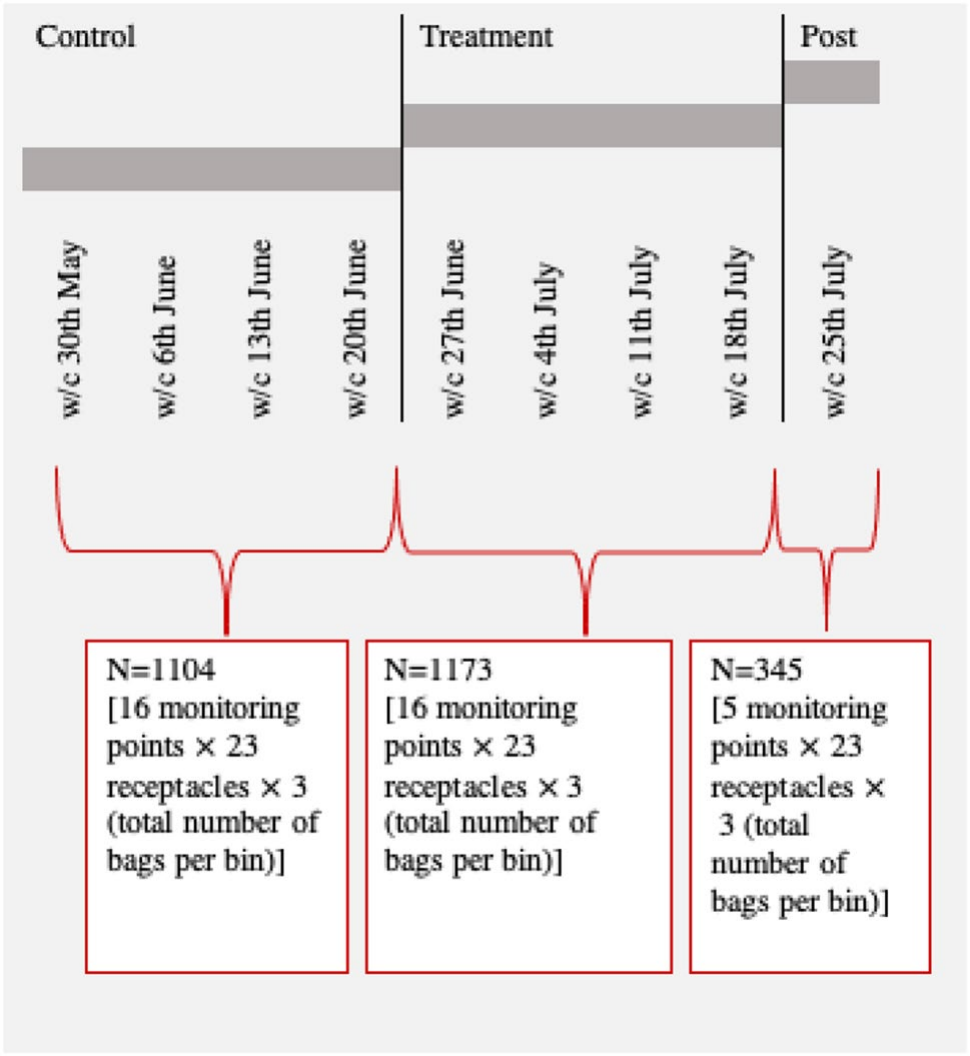
Experimental timeline.

Primary data was collected on weekdays after working hours (Monday to Friday from 5 to 7 pm). This time was selected to reduce as much as possible contact with participants and preserve the study validity from data contamination. Data was collected from bin receptacles containing the three bags – non-recycling, mixed recycling, and food waste – as shown in Fig. 1, panel (a). Each bin was removed from the receptacle, weighted, and carefully scrutinised to identify sorting errors (see section “Defining sorting errors” below). Transparent plastic bags facilitated this process making it possible to identify waste inappropriately sorted. We recorded information on total bin’s weight and errors per bin per day. These figures were then used for the empirical analysis detailed below in section “Results”. Data collection took place for a period of 9 weeks between May and July 2022. The pre-treatment monitoring period occurred between w/c 30th May and w/c 20th June and involved 16 audits, on 23 bin receptacles that were weighed 368 times for a total of 1104 weight data points. The treatment period took place between w/c 27th June and w/c 18th July, and included 17 audits on 23 waste disposal bins, weighed 391 times for a total of 1173 observations. Finally, a short post-treatment period was completed during w/c 25th July and included 5 audits, on 23 bin receptacles, weighed 115 times giving a total of 345 observations. Throughout the study a total of 38 audits were conducted for each of the 23 bin receptacles selected for the experiment, giving the total of 2622 observations used in the empirical analysis described below (see section “Empirical strategy”). The experimental timeline is presented as Fig. 2, while Table 1 lists the receptacles by building and floor.

The pre-treatment instructions (kept in our control group, CH, and in the first treatment) were already present in the buildings. As illustrated in Fig. 1 (see panel b), visual and written instructions were referred to non-recycling, mixed recycling, and food waste, providing some information on how recyclables could be utilised if sorted correctly.

**Figure 3:**
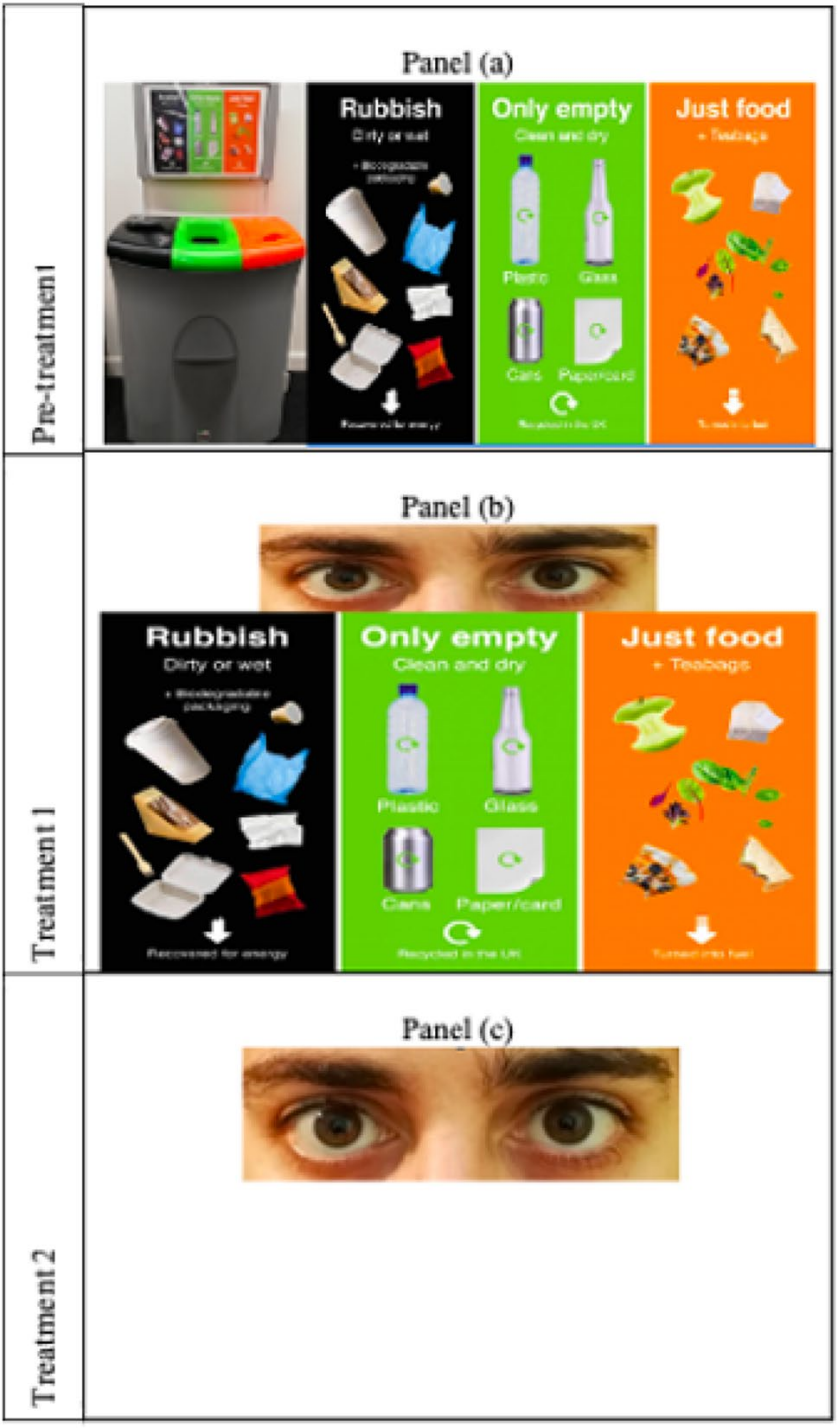
Manipulation of the two interventions.

The posters displaying the interventions are shown in Fig. 3. For the watching eyes (Fig. 3, panel (b)), we follow the literature and consider the open, and serious expression of a male pair of human eyes (see Ernest-Jones et al*.*^16^; and Bateson et al.^19^). The visual nudge is then combined with pre-existing recycling visual instructions placed on top of the bins. The objective of the visual nudge here is to attract individuals’ (limited) attention and re-direct it towards the instructions, fostering prosocial behaviour via an injunctive social norm (i.e. by reinforcing the message of explicit verbal and visual prompts).

The study allows a viable comparison with the effect of the eye-nudge without the pre-existing instructions (Fig. 3, panel (c)), to test whether recycling behaviour can be triggered by reputation-based cooperation alone. Finally, by removing the treatments and replacing only the pre-existing instruction, we look at short-lived effects.

### Defining sorting errors

In the non-recycling, sorting errors were identified as clean items not placed in the mixed-recycling bin receptacles (thus becoming dirty) or as food residuals present in the bags. In the mixed-recycling error items were categorised as items placed in the mixed-recycling bags different from plastic material, papers, cardboard, cans, or glass, or as dirty or wet mixed recycled material. We categorised items as ‘dirty’ when undisputedly not washed or rinsed before being placed into the bin. Items were deemed ‘clean’ when no heavy stains and/or high contamination was present. Food residuals were also considered as sorting errors when items were erroneously placed in the mixed-recycling bins. In the food waste, sorting errors consisted of items placed in the food waste bags different from food waste or tea bags. Error clusters and cluster codes utilised for data analysis are detailed in Appendix (see Tables A1 and A2). This study disregarded empty bins, excluding them from the analysis, as they would have caused inaccurate results by not differentiating between empty bins and bins with no sorting mistakes.

## Empirical strategy

A balanced panel regression Difference-in-Differences (DiD) model was adopted to measure the effectiveness of the two treatments. The regression investigates interactions and extra-trial factors which might influence the results. Finally, heatmaps were adopted for visual analysis to investigate the differences in sorting efficacy between clusters.

### Parameter of sorting behaviour

Our initial point of reference is the food waste methodology presented by Barker et al*.*^27^, identifying the percentage of error for each bin as an appropriate indicator of sorting behaviour.

We compute the sum of the bins’ error weight ${B}_{er}$, obtained by multiplying the number of mistakes identified, ${N}_{m}$, by the respective average error cluster weight, ${W}_{m}$. Average weights for each error type/cluster are detailed in Table A1 in the Appendix. We then calculate the percentage error ratio, ${E}_{r}$, derived by dividing the bins’ error weight ${B}_{er}$ by the total bin weight ${W}_{b}$, multiplied by 100. We formulate this as follows:

1 $${B}_{er }= {\sum }_{m=1}^{M}{N}_{m}\cdot {W}_{m},$$

2 $${E}_{r}=\frac{{B}_{er}}{{W}_{b}} \cdot 100,$$

were, $m$ represents the error types and varies between $1-19$, i.e. $m(1\dots 19)$.

The percentage indicator for each bin is also used to estimate how much recyclable waste is re-directed to incinerators or landfills due to contamination. To estimate the amount of actual recycled and re-directed waste we employ a tolerance factor, $\tau ,$ of 10% and 50% for mixed-recycling and 50% food waste, respectively. As shown in Eq. (3), we compute the percentage of non-recycled bins, ${R}_{b\%}$, by dividing the sum of the unrecycled (re-directed) bins, ${U}_{b}$, over the total number of bins, ${N}_{b}$:

3 $${R}_{b\%}= \frac{\sum {U}_{b}}{{N}_{b}}.$$

The tolerance factor $\tau$ is used therefore to determine ${U}_{b}$. Specifically, bins are classified as unrecycled (re-directed) bins if the percentage of non-recycled waste is greater than $\tau$, that is when ${E}_{r}>\tau$.

### The model

A balanced DiD panel regression test was adopted to analyse the 759 observations of 23 receptacles over 33 days. By comparing the control to the treatment group within the given time period, we aim to identify any associated changes in recycling errors to the treatment effect, thus excluding ‘noise’ due to other external factors. Following Abadie and Cattaneo^28^, the model can be summarised as follow:

4 $${Y}_{it}={\alpha }_{it}+ {\beta }_{1}\cdot {TREAT}_{t} + {\beta }_{2}\cdot {POST}_{i}+ {\beta }_{3}\cdot \left({TREAT}_{t}\cdot {POST}_{i}\right)+ {\gamma }_{iT}\cdot {X}^{^{\prime}}+ {\varepsilon }_{it}.$$

${Y}_{it}$ indicates the percentage of sorting errors for a selected bin – non-recycling, mixed-recycling, food waste. $i$ and $t$ are respectively the indicators of the receptacle’s identity and time. ${\alpha }_{it}$ is a constant. ${\beta }_{1}$ represents the regression coefficient for the pre-treatment differences, that is the baseline differences between control and treatment groups. TREAT_t_ is a dummy variable taking values 1 if a bin belongs to the treated group, and 0 otherwise. POST_i_ is a dummy for post-treatment periods, which equals 1 for the treatment period, and 0 for pre-treatment. Thus, ${\beta }_{2}$ captures the fact that conditions change over time across groups. ${\beta }_{3}$ is the time- and group-invariant coefficient that controls for differences in time periods across groups –i.e. the Difference-in-Difference (DiD) causal effect. ${\gamma }_{it} \cdot {X}^{^{\prime}}$ examines the effect that external factors might have on sorting behaviour, such as the floor level at which receptacles are placed and their location, the sample population, the weekday, and the presence of extra-trial receptacles in proximity. ${\varepsilon }_{it}$ is an error term.

Considering the percentage of sorting errors, the DiD coefficient, ${\beta }_{3}$, is computed by subtracting the average errors in the control group from the average errors in the treated groups, as shown by Eq. (5):

5 $$DiD\left({TREAT}_{t}\cdot {POST}_{i}\right)=\left(T\left[i,tx\right]-C\left[i,pre\right]\right)- \left(C\left[tx\right]-C\left[pre\right]\right),$$

with $i=\mathrm{1,2}$, and $T[i,tx]$ and $C[i,pre]$ representing respectively average errors in treated groups during the treatment period, and pre-treatment period; and $C[tx]$ and $C[pre]$ capturing average errors in the control group during the treatment and pre-treatment period, respectively (see Table 2 below). Results are referred to using the terminology detailed in Table 2, which shows the label of the different groups, their time periods and whether they had an intervention.

**Table 2:** Group label.

Label	Time-period	Intervention
T[1,pre]	Pre-treatment	No
T[1,tx]	Treatment	Treatment 1
T[1,post]	Post-treatment	No
T[2,pre]	Pre-treatment	No
T[2,tx]	Treatment	Treatment 2
T[2,post]	Post-treatment	No
C[pre]	Pre-treatment	No
C[tx]	Treatment	No
C[post]	Post-treatment	No

## Results

We first look at baseline average weights to check whether a viable comparison across different sites is possible. As shown in Table 3, overall, the pre-treatment groups show similar averages of waste per bin (p > 0.1). However, the difference in waste weight between the control and pre-treatment groups in treatment1 and that of the control and pre-treatment groups in treatment 2 are statistically significant (at 1% and 5% significance level, respectively). A close scrutiny of data reveals that the former might be attributable to the type of waste generated in non-recycling bins, where a large majority of mistakes are due to misplaced food waste. The latter can be attributed to a high number of empty food bins, whose difference is more balanced when comparing the control and pre-treatment groups in treatment 1 (≈50% in both groups), but becomes stark when considering the same two groups in treatment 2 (52% in the control group vs 64% in treatment 2). We speculate that this may be due to differences in the number of people circulating in the buildings during data collection. It is also worth noting that treatment 2 was only tested on five receptacles as compared to the nine considered in the control and treatment 1 groups.

**Table 3:** Baseline average waste across treatments (grams per weekday).

Bins	Weight (g)/C[pre]	Weight (g)/T[1,pre]	Diff [C[pre]-T[1,pre]]	Weight (g)/T[2,pre]	Diff[C[pre]-T[2,pre]]
Non-recycling	170.83	239.79	− 68.95***	202.25	− 31.41
			(0.002)		(0.202)
Mixed-recycling	267.64	328.96	− 61.31	306.88	− 39.26
			(0.101)		(0.357)
Food recycling	140.49	128.33	12.15	57.62	82.86**
			(0.705)		(0.013)

The table reports average waste across pre-treatments sites, their differences and independent samples *t* tests of which *p*-values are reported in parentheses.

### Trends

**Figure 4:**
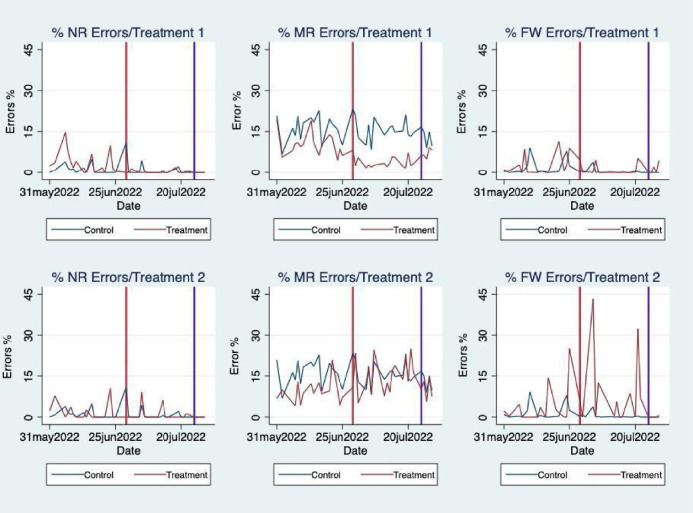
Trends of percentage errors per treatment. The table reports the % of recycling errors for each bin considered in the analysis. The vertical red line in the diagrams indicates the date in which the treatment was introduced (i.e. 29 June 2022), whereas the blue line indicates the post intervention date (i.e. 25 July 2022).

Graphic trends of the percentage of recycling errors can be found in Fig. 4 for treatments 1 and 2 (i.e. non-recycling, mixed-recycling and food waste). The vertical lines represent the policy interventions (i.e. treatment and post-treatment time periods, respectively) at day seventeen and thirty-four of the experiment. Looking at the top panel of the figure, our results show that after the implementation of the treatment, the effect on mixed-recycling bins is to significantly decrease errors, which start to increase again during the post-treatment. Interestingly, in the other two bins, the errors stabilise at a lower level during the treatment period and there is no significant effect of removing the intervention. The bottom panel of the figure focuses on treatment 2, where, overall, we see that, during the treatment, errors tend to increase especially for food and not recycling waste.

### Difference-in-differences model

The average percentage of sorting errors is reported in Table 4, which summarises differences between pre-treatment and treatment periods by groups. Results suggest that treatment 1 generated a reduction of percentage errors in all bins, while the opposite seems to occur in treatment 2 for mixed-recycling and food waste. On average, in treatment 1, percentage errors decline by 64%, 95%, and 58%, respectively in mixed-recycling, non-recycling, and food waste bins. Control bins display similar percentage errors across different conditions.

**Table 4:** Average percentage of sorting errors.

	NR %	MR %	FW %
T[1,pre]	3.41	10.37	2.12
	(0.82)	(0.91)	(0.74)
T[1,tx]	0.14	3.69	0.89
	(0.10)	(0.44)	(0.37)
T[2,pre]	1.92	8.59	2.42
	(0.86)	(1.13)	(1.04)
T[2,tx]	0.91	13.59	5.06
	(0.51)	(1.40)	(2.02)
C[pre]	1.05	13.43	1.77
	(0.40)	(1.16)	(0.54)
C[tx]	0.86	13.80	0.31
	(0.40)	(0.89)	(0.15)

The table reports average errors per bins (%), standard errors are reported in parentheses.

Tables 5 and 6 report the econometric results of the regressions, the first one referring to treatment 1 and the second considering the eye nudge without providing information on sorting waste.

**Table 5:** Treatment regressions 1 results.

	(1)	(2)	(3)	(4)	(5)	(6)
	NR recycling	NR recycling	MR recycling	MR recycling	FW recycling	FW recycling
	Errors (%)	Errors (%)	Errors (%)	Errors (%)	Errors (%)	Errors (%)
TREAT_1 X POST	− 3.235**	− 3.081**	− 6.924***	− 7.197***	0.138	0.211
	(1.045)	(0.959)	(1.006)	(1.301)	(0.797)	(0.801)
TREAT_1	1.055		− 1.489		1.658*	
	(0.941)		(2.596)		(0.885)	
POST	− 0.190		0.240		− 1.443**	
	(0.376)		(0.755)		(0.454)	
Floor number	0.075	0.150	0.448	0.374	0.241*	0.300**
	(0.248)	(0.260)	(0.576)	(0.546)	(0.128)	(0.131)
Extra-trial bins	− 0.937	− 1.125	1.500	1.708	0.823*	0.494
	(0.879)	(0.945)	(2.154)	(1.926)	(0.413)	(0.475)
Location						
(Base category: hallway)						
Kitchen/corridor	2.325**	2.541**	0.085	− 0.084	− 2.271**	− 1.565*
	(0.855)	(0.874)	(2.440)	(2.089)	(0.795)	(0.766)
Room	0.657	0.135	0.347	1.426	0.126	− 0.248
	(0.802)	(1.202)	(1.258)	(2.090)	(0.582)	(0.836)
Kitchen	0.971	0.278	− 0.221	0.842	− 0.874	− 1.775
	(1.131)	(1.451)	(2.958)	(3.177)	(0.589)	(1.144)
Library	− 0.656	− 1.291	12.807***	13.878***	− 0.102	− 1.393
	(0.785)	(1.064)	(2.811)	(2.549)	(0.501)	(0.884)
Participants						
(Base category: mixed)						
Students	− 0.520	− 0.625	− 2.442	− 2.484	1.719**	1.536**
	(0.933)	(0.982)	(1.490)	(1.621)	(0.461)	(0.532)
Staff	0.070	0.018	2.954	2.951	0.813*	0.680
	(0.862)	(0.931)	(1.739)	(1.891)	(0.429)	(0.539)
Constant	1.177	1.713	9.062**	8.217**	− 0.158	0.275
	(1.201)	(1.356)	(2.531)	(2.370)	(0.413)	(0.643)
Observations	478	478	520	520	301	301

Standard errors clustered at (unique) Bin ID in parentheses. All models report time (date of data collection) and weekday fixed effects.

The variable *extra trial bins* takes values 1 = if there are extra trial factors, and 0 = otherwise. The variable *floor number* represents floor numbers and takes values between 1(ground floor) and 8 (sixth floor).

*p < 0.1, **p < 0.05, ***p < 0.001.

**Table 6:** Treatment 2 regressions results.

	(1)	(2)	(3)	(4)	(5)	(6)
	NR recycling	NR recycling	MR recycling	MR recycling	FW recycling	FW recycling
	Errors (%)	Errors (%)	Errors (%)	Errors (%)	Errors (%)	Errors (%)
TREAT_1 X POST	− 0.591	− 0.357	4.588**	4.436**	3.721**	3.931**
	(0.803)	(0.824)	(1.796)	(1.969)	(1.158)	(1.425)
TREAT_1	1.121		− 1.123		− 3.601	
	(1.778)		(4.253)		(2.385)	
POST	− 0.169		0.184		− 1.364**	
	(0.378)		(0.775)		(0.455)	
Floor number	0.474*	0.636**	− 1.254	− 1.351**	0.707**	0.422*
	(0.248)	(0.171)	(0.862)	(0.525)	(0.263)	(0.238)
Extra-trial bins	0.483	0.198	− 3.353**	− 3.374**	1.314	1.526
	(0.657)	(0.662)	(1.192)	(1.497)	(2.447)	(1.842)
Location						
(Base category: hallway)						
Kitchen/corridor	− 4.649**	− 4.743***	12.253***	12.262***	2.908***	0.328
	(1.231)	(1.091)	(1.398)	(1.739)	(0.660)	(1.887)
Room	− 0.282***	− 0.107	1.654***	2.237**	0.640***	1.650
	(0.004)	(0.516)	(0.066)	(0.822)	(0.038)	(1.548)
Kitchen	− 1.339**	− 1.712**	3.700	4.053	0.651	− 0.833
	(0.496)	(0.556)	(2.925)	(2.999)	(1.045)	(2.663)
Library	− 1.423**	− 1.874**	12.601***	13.280***	2.476	2.036
	(0.642)	(0.640)	(1.124)	(1.691)	(2.363)	(1.456)
Participants						
(Base category: mixed)						
Students	1.669***	2.066***	− 7.094***	− 7.199***	− 0.372	0.907
	(0.248)	(0.412)	(0.853)	(0.727)	(0.276)	(1.422)
Staff	1.208**	1.351**	4.656	5.125	− 1.881	0.627
	(0.411)	(0.549)	(3.080)	(3.208)	(1.088)	(1.726)
Constant	− 1.051	− 1.394	17.351***	17.211***	− 1.608	− 2.390
	(0.937)	(1.023)	(2.734)	(3.056)	(2.702)	(2.018)
Observations	359	359	379	379	200	200

Standard errors clustered at (unique) Bin ID in parentheses. All models report time (date of data collection) and weekday fixed effects. The variable *extra trial bins* takes values 1 = if there are extra trial factors, and 0 = otherwise. The variable *floor number* represents floor numbers and takes values between 1(ground floor) and 8 (sixth floor).

*p < 0.1, **p < 0.05, ***p < 0.001.

Results reported in Table 5 confirm the effect of Treatment 1, with a reduction in error weight percentage for non-recycling and mixed-recycling of 3.2 and 6.9 percentage points, respectively (both significant at p < 0.01). Regarding the other independent variables, floor number and having extra-trial bins do not seem to have significant effects, except for food waste where, as expected, they increase the percentage of mistakes. Mixed-recycling bins exhibited significantly higher mistakes in the library, while, considering food waste bins, the percentage error was higher when the surrounding area was accessed mainly by students (at 5% significance level). Table 6 shows that treatment 2 significantly affects the percentage of errors which increase by 4.5 and 3.7 percentage points, respectively for mixed-recycling and food waste (both at 5% significance level). Higher floors are significantly correlated with higher mistakes for not-recycling and food waste bins. Compared to the hallway location, other areas recorded significantly higher percentages of mistakes for mixed recycling and food waste (kitchen excluded), though they seem to decrease for not-recycling bins. With the eye nudge placed without providing the additional recycling instructions, students made significantly lower mistakes in the mixed-recycling, while these were slightly higher in the non-recycling bins (both at 1% significance level). Similarly, members of staff were more incline to make mistakes in non-recycling bins, with mixed-recycling showing a similar pattern of behaviour (though not statistically significant).

**Figure 5:**
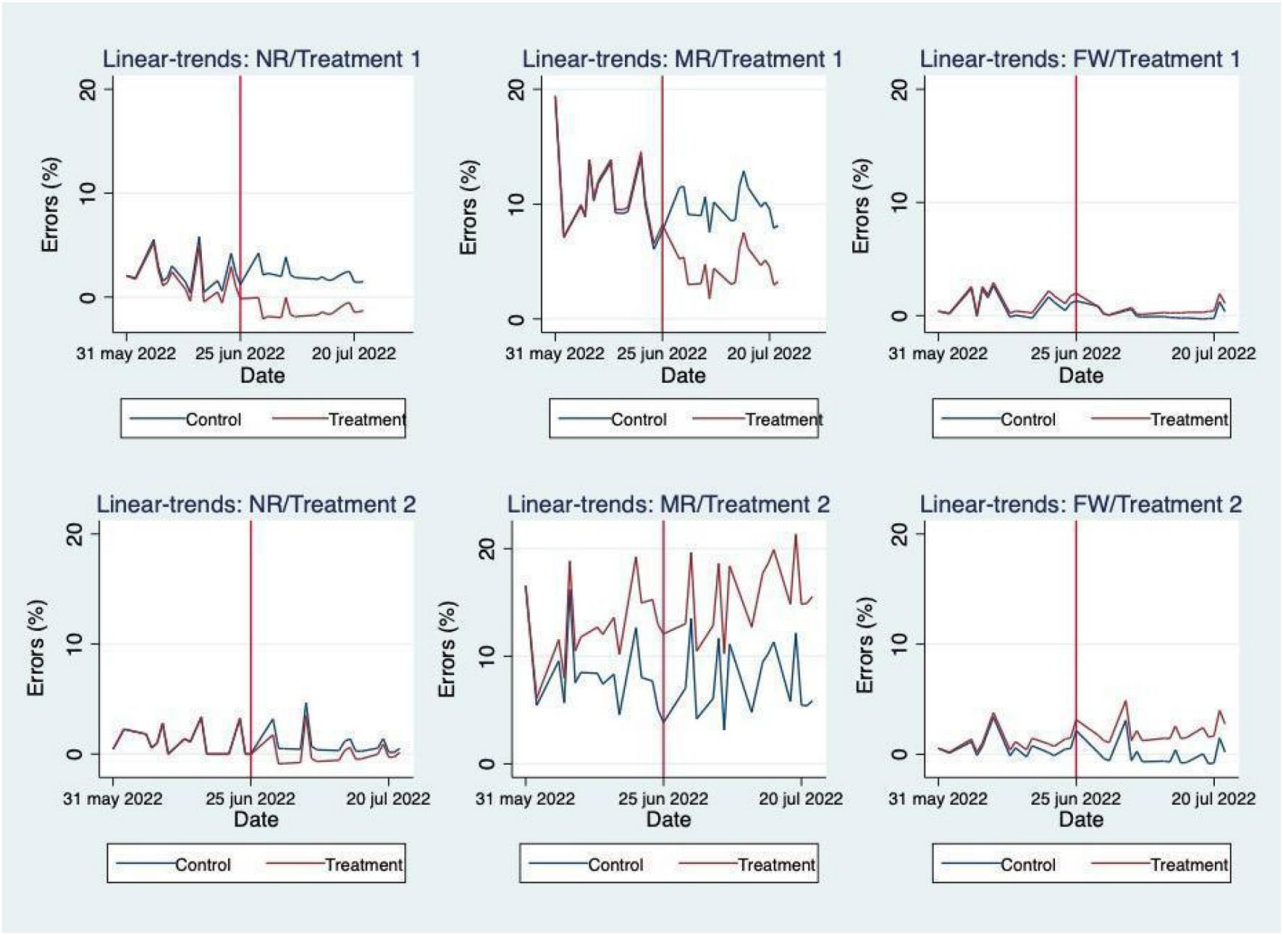
Linear trends.

A parallel trend visual test successfully reveals that the model assumptions are verified (Fig. 5).

### Heatmap analysis

**Figure 6:**
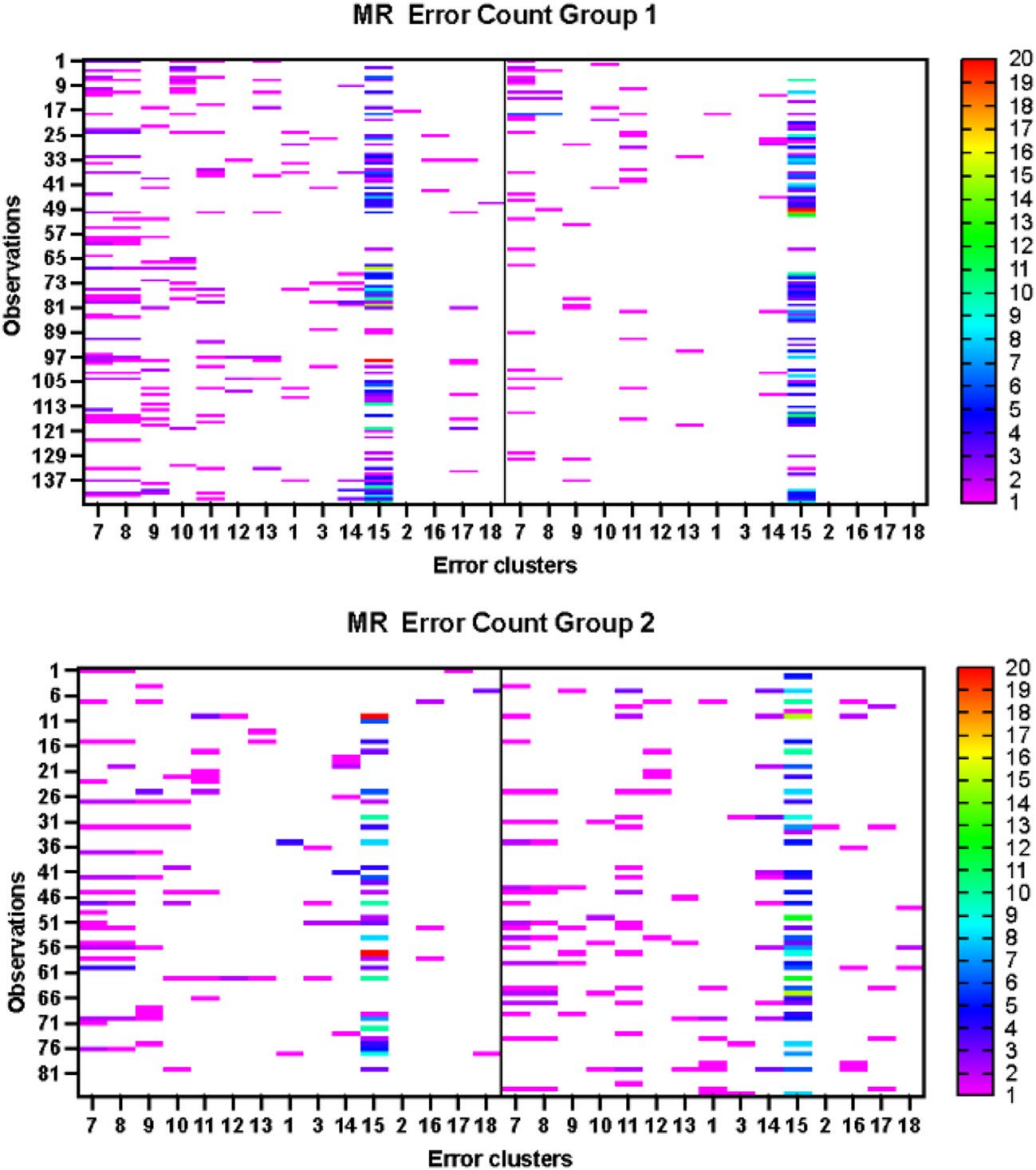
Heatmaps of sorting errors.

To investigate the differences in sorting efficacy between clusters, we produce a visual representation of the sorting error clusters. For this section and the following one, we focus our analysis on mixed-recycling bins, as the baseline comparisons reveal that these bins show consistent similar characteristics across treatment conditions (see Table 3). In Fig. 6, the error clusters are represented on the X axis and single observations on the Y axis. Heatmap analysis is conducted for treatments 1 and 2 (in the table groups 1 and 2) between control (pre-treatment, $T[i,pre]$) and treatment periods ($T[i,tx]$), illustrated respectively in the left and right quadrant of each heatmap provided below. The number of mistakes reported in the table is taking values 0 to 20, which are reported using different colours in a scale from white to red. The coloured bar on the right-hand side of the tables reports the scale and colours of each error cluster. In group 1, results confirm a reduction in number of mistakes moving from $T[1,pre]$ to $T[1,tx]$. This is shown in the diagram by the increased number of white cells present on the right-hand side. However, this is not homogeneous within clusters. During the treatment period, food-related errors seem to disappear (see cluster codes 16,17,18 on the x-axis), whereas other errors (such as coffee cups (cluster code: 7) and tissues (cluster code: 15) tend to persist. Different is the situation when considering group 2 (see bottom panel of the diagram), where overall there is evidence of increasing mistakes moving from $T[2,pre]$ to $T[2,tx]$.

### Tolerance analysis

**Figure 7:**
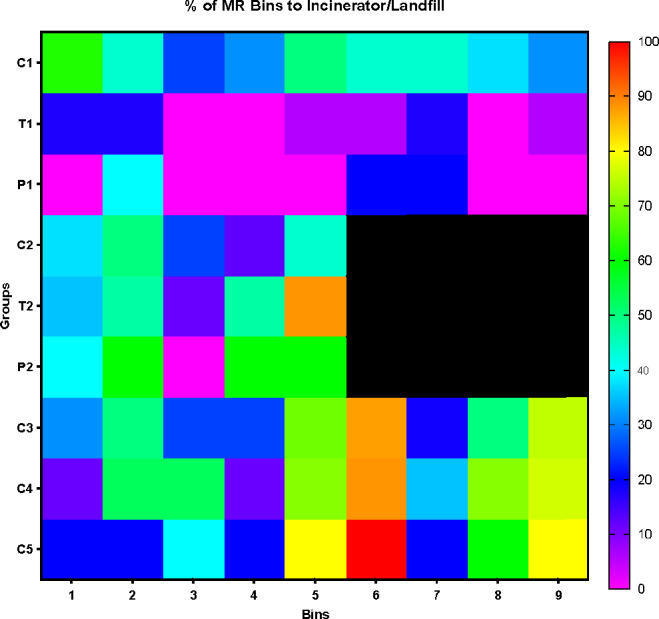
Heatmap analysis of re-directed bins to incinerators or landfill. C1, T1 and P1 represents respectively the pre-, during, and post- treatment conditions of the groups. Similarly, C2, T2 and P2 indicate treatment conditions for group 2 and C3, C4 and C5 are used for the control group.

The tolerance factor described in section “Parameter of sorting behaviour” allows us to better understand how much waste is (or is likely to be) sent to an incinerator and/or landfills. For this analysis, the empty bins were not discarded, as they are usually not sent to incinerators/landfills. In addition, bin usage varies between locations, thus removing some of them from the analysis would have led us to misleading results and conclusions. This is to say that, over a total of 16 observations, a mixed-recycling bin used only once with a percentage error ratio higher than 10% (${E}_{r}>\tau$, where $\tau =10\%$) would be equivalently compared to one used 16 times with a similar percentage error $({E}_{r}>10\%)$ each time. Data is shown in the heatmap in Fig. 7, which focuses on mixed recycling: each row represents a treatment, while columns report bins’ information. Each square is coloured according to a scale representing the number of times a bin goes to incinerators/landfill after an audit. The heatmap shows that groups $C[pre]$, $C[tx]$ and $C[post]$ are homogeneous, and bins 5, 6, 8, and 9 have a higher percentage of waste sent to incinerators/landfills. In general, as treatment 1 is likely to reduce the number of mistakes, it also leads to a low rate of bins aimed at incinerators/landfill (between 0 and 20%). This is illustrated in the diagram by the range of cells coloured in blue and purple. Except for bin 5, located in the library and therefore mainly exposed to the student population, bins 6, 8 and 9 were all located in the staff kitchen. Regression results (see Table 6) suggest that there is a significant and positive effect of population on percentage of errors which seems to be confirmed by the heatmap analysis. It is striking the purple area around $T[1,tx]$ which shows the effectiveness of treatment 1. The black area is due to the lower number of bins present in treatment 2 as compared to the other two treatment conditions.

### Post-treatment analysis

To evaluate sorting behaviours after the treatment period we conduct an Anova analysis to test differences in recycling behaviour (average percentage errors) across different treatment conditions once the treatment was removed. Results are reported in the Appendix. To have a balanced dataset, the analysis considered the first 5 days of pre-treatment and treatment periods, as the post-treatment only had five observations. Our findings suggest that, in treatment 1, mixed-recycling bins report a strong significant difference in percentage errors when the visual nudge is removed (p < 0.01, see Table A3 in Appendix), increasing mistakes from 3.69 to 7.74% ($\pm$ 1.28 SE). However, results seem to confirm no significant differences when comparing treatment and post-treatment bins in all other groups.

## Discussion of results and conclusions

To the best of the authors’ knowledge, this is the first study in the literature that looks at the causal relationship of visual nudges and information on recycling behaviour. The most significant results were seen in treatment 1, where we combined (visual and easy-to-grasp written) instructions with watching eyes. As discussed in section “Results”, the DiD analysis shows that the combination of these two interventions caused a significant decrease in errors for non-recycling and mixed recycling by a large amount. At the same time, having the eye nudge without information in treatment 2 increased sorting errors for mixed recycling and food waste. It is interesting to note that here students were less likely to make mistakes when considering mixed recycling, while both students and staff seemed more likely to make mistakes when sorting waste in non-recycling bins.

Therefore, results are in line with existing literature showing that subtle cues of observation affect individuals’ behaviour (Van Doesum et al.^29^). However, we speculate that, in our study, this result is more likely to be driven by enhanced attention towards recycling instructions rather than to reputational loss (see e.g. Ernest-jones et al.^16^, Kawamura and Kusumi^30^). In the literature on littering, the effect of watching eyes alone is generally stronger than other types of interventions (e.g. financial intervention, norm-based intervention, ecology-based intervention, or eye images combined with verbal instructions). In line with this literature, if reputational loss plays a major rule here, we expect to observe a stronger reduction in errors when considering the eye nudge alone. However, our results not only suggest that eye-nudges combined with verbal instructions are superior in improving recycling behaviour (as compared to treatment 2 and the control group). Differently from other research on littering, our analysis also shows that, overall, the percentage of sorting errors in treatment 2 is higher than that observed in treatment 1 and the control group, which suggests that removing instructions tends to increase confusion on how to sort waste. Conversely, when information is provided, the eyes capture individuals’ attention to recycling instructions thus reducing sorting errors.

Our findings also uncovered error clusters differences across different treatments. The heatmap analysis suggests in fact that, in treatment 1 mixed-recycling bins, food errors almost vanished, while the most persistent mistakes were coffee cups and tissues. It is difficult to disentangle reasons for incorrect sorting behaviour. This could be due, for example, to confusion on how to sort waste, and/or negligence. Our data does not allow us to make clear conclusions on this finding.

The tolerance heatmaps also shows that food errors significantly improved, from $T[1,pre]$, where bins were frequently sent to incinerator/landfill (30–60% of the time) to $T[1,tx]$, where bins were sent to incinerator/landfill no more than 20% of the time. Bins number 1, 2, and 7 are those that were more often sent to landfill (19% of the time). Assuming a homogeneous effect on all bins across campus and considering that in $T[1,pre]$ there were approximately 3 kg of mixed-recycling collected per bin per week, we estimate a decrease of waste re-directed to incinerators or landfill equivalent to 15.6–62.4 kg per mixed-recycling bin per year. If we extend the effect of the treatment condition to the 23 bin receptacles included in our experiment, this means saving between 358.8 and 1435.2 kg of waste re-directed to incinerators or landfill.

Regarding treatment 2, our analysis shows that, in the absence of detailed instructions, recycling rates decrease, making waste management more complicated, with more persistent errors that do not show a clear pattern. From a behavioural perspective, an important aspect to consider is that ‘recycling rules’ are often different depending on country, municipality, and site. Standardisation of waste collection policies in England has been recently suggested by the UK government as a possible solution to reduce chaos and confusion around recyclable waste materials sorted by households and businesses. The lack of consistent waste collection policies makes it harder for people to form common recycling habits as recycling collection rules are context dependent. This leads people to make careless and unintentional mistakes, which may be exacerbated in a multicultural context such as a university campus and by the lack of instruction in treatment 2.

It is also worth noting that our study does not differentiate between clean and dirty misplaced items. This differentiation was extremely hard to make, as it is not possible to check whether a dirty item was placed in the bin because it was dirty or became so due to individuals’ incorrect sorting behaviour. We noted that in treatment 1 bins were generally cleaner, and items such as coffee cups, or paper lunch containers were rinsed before being placed in the bins. From a recycling perspective, such items are not properly sorted due to contamination errors. However, from a behavioural perspective this suggests a change in behaviour which might be triggered by a simple and unconscious mechanism such as a nudge (i.e. in the form of eye images). While a scientific conclusion cannot be drawn due to the difficulties in data collection, we hypothesise that such behaviour could be due to confusion or overconfidence on how to sort waste rather than negligence. Further research might help disentangle the different reasons that motivate sorting behaviour; at the same time, the post-treatment observation period was too limited to bring conclusive evidence on the long-term effects of these nudges.

On generalisability, the experiment was conducted with a highly educated population, who was likely to be aware of the compounding effects of not recycling. Bins were also well designed, and the instructions clear and salient. In future research, it would be interesting to test whether these findings hold within different contexts.

Our research provides additional evidence on the relevance and effectiveness of low-cost interventions and easy to implement behavioural tools to change individuals’ actual sorting behaviour. Stimulating individuals’ attention towards (visual and easy-to-grasp written) instructions can help them engage more with recycling and thus reduce recycling mistakes. Our interventions build upon a vast literature largely focused on littering showing the effectiveness of watching eyes as a behavioural tool to boost pro-environmental behaviour in controlled and natural contexts. However, differently from this literature, our analysis suggests that results can be explained by attention-based (rather than reputation-based) motivations. The complexity of recycling might explain our findings.

In line with the most recent literature on nudging interventions, our analysis provides some indications of the effect of removing nudges highlighting their short-lived effect.

Looking at the limitations of this study, the design of our experiment only allows us to speculate about possible mechanisms driving individuals’ decision-making. Unfortunately, our data does not allow us to draw any conclusions about the working mechanism of the nudge intervention. Data on bins’ weight and sorting errors is measured at aggregate level considering information on bags placed in each of the bins considered in the study. Thus, we can only infer the average treatment effect at aggregate level. Our dataset does not contain information on events that happened in specific days and floors, as well as on the number of individuals entering the buildings in the treated areas. We could not test the effect of different types of eyes, which may help drawing conclusions on the impact of positive framing, gender, and ethnicity. It would be interesting to see the results of our analysis replicated and extended in further research. To answer the question in the title of this paper and to pun the speaker of the house of commons’ announcement when a legislative motion is passed “the ‘eyes’ have it” but not in isolation.

## Supplementary Information

Supplementary Tables.

## Data Availability

The datasets used and/or analysed during the current study available from the corresponding author on reasonable request.
