# Supplementary material for: Improving recycling sorting behaviour with human eye nudges
Source: Sci Rep. 2023 Jun 22;13:10127. doi: 10.1038/s41598-023-37019-x (PMC10287684; doi:10.1038/s41598-023-37019-x)
Supplement: Supplementary file 1 — Supplementary Tables. [file 41598_2023_37019_MOESM1_ESM.docx]

**Appendix**

Table A1: Average weight error cluster

| Misplaced or Clean |  | Misplaced or dirty |  | Misplaced |  |
| --- | --- | --- | --- | --- | --- |
| NR | (g) | MR | (g) | FW | (g) |
| Plastic container | 25 | Plastic container | 25 | Plastic container | 25 |
| Paper bag | 30 | Paper bag | 30 | Paper bag | 30 |
| Plastic Bottle (Water) | 15 | Plastic bottle (Juice) | 20 | Paper tissue | 1 |
| Cans | 12 | Plastic cup | 15 | Metallised plastic | 2 |
| Glass Bottle | 225 | Plastic lid | 5 | Plastic cup | 20 |
| Food Waste | 43 | Plastic glass | 20 |  |  |
| Tea bags | 5 | Paper lunch box | 20 |  |  |
|  |  | Sandwich box | 15 |  |  |
|  |  | Specific brand lunch box | 65 |  |  |
|  |  | Soup box | 20 |  |  |
|  |  | Metallised plastic | 2 |  |  |
|  |  | Paper tissue | 1 |  |  |
|  |  | Banana | 45 |  |  |
|  |  | Sandwich | 40 |  |  |
|  |  | Tea bag | 5 |  |  |

*Notes*: The average weight of each error cluster type, $W_{m}$ in equation (1), is calculated counting 10 items per type of error cluster and averaging their weight.

Table A2: Error cluster codes

| Cluster code | Error cluster |
| --- | --- |
| 1 | Plastic container |
| 2 | Paper bag |
| 3 | Plastic bottle (Juice) |
| 4 | Plastic Bottle (Water) |
| 5 | Cans |
| 6 | Glass Bottle |
| 7 | Plastic cup |
| 8 | Plastic lid |
| 9 | Plastic glass |
| 10 | Paper lunch box |
| 11 | Sandwich box |
| 12 | Specific brand lunch box |
| 13 | Soup box |
| 14 | Metallised plastic |
| 15 | Paper tissue |
| 16 | Banana |
| 17 | Sandwich |
| 18 | Tea bag |
| 19 | Food Waste |

Table A3: Two-way Anova analysis

| Treatment | Pairwise comparison | NR | MR | FW |
| --- | --- | --- | --- | --- |
| 1 | CTP | 0.053* | 0.001 *** | no |
| 1 | CT | 0.049** | 0.002*** | no |
| 1 | TP | no | no | no |
| 2 | CTP | no | no | no |
| 2 | CT | no | 0.06 | no |
| 2 | TP | no | no | no |
| control | CTP | no | no | no |
| control | CT | no | no | no |
| control | TP | no | no | no |

*Notes:* The table reports t-tests results in Treatment 1, with CTP (which tests average percentage errors in treatment condition versus pre-treatment and post-treatment); CT (which tests average percentage errors in treatment condition versus pre-treatment); and TP (which tests average percentage errors in treatment condition and post-treatment condition).

****p<0.001, ***p<0.01, **p<0.05, *p<0.1, “no” = no significant value
